# Supplementary figures and images for: Recognition of apnea and hypopnea by non-contact optical fiber mattress and its application in the diagnosis of obstructive sleep apnea hypopnea syndrome: a retrospective study
Source: PeerJ. 2024 Jun 17;12:e17570. doi: 10.7717/peerj.17570 (PMC11188932; doi:10.7717/peerj.17570)

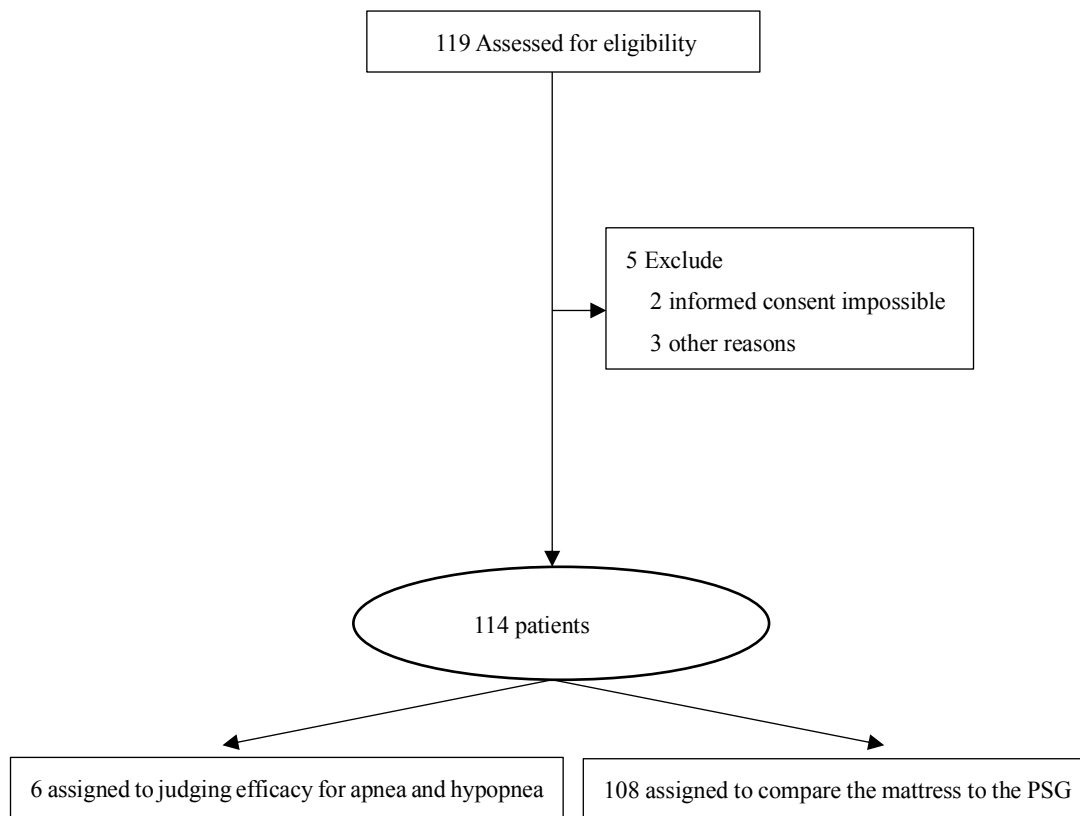

Supplement: Supplemental Information 1 [file peerj-12-17570-s001.pdf]
